# Cellular senescence in ovarian cancer: a proposed geroncogenic shift linking aging, plasticity, and therapy resistance

**DOI:** 10.3389/fphar.2026.1972486

**Published:** 2026-10-08

**Authors:** Yubo Zhang, Yufei Han, Ping Hou, Weina Zhang, Meng Wu, Mengqi Deng

**Affiliations:** 1 Qingdao Hospital, University of Health and Rehabilitation Sciences (Qingdao Municipal Hospital), Qingdao, China; 2 Beijing Obstetrics and Gynecology Hospital, Beijing Maternal and Child Healthcare Hospital, Capital Medical University, Beijing, China

**Keywords:** aging, cancer stem cells, cellular senescence, geroncogenic shift, ovarian cancer, senescence-associated secretory phenotype, senolytics, therapeutic resistance

## Abstract

Ovarian cancer develops within a biological environment that changes substantially with age. Beyond chronological aging, progressive alterations in genome maintenance, telomere integrity, epigenetic regulation, mitochondrial function, metabolism, inflammation, and immune surveillance can reshape the cellular and tissue context in which tumor evolution occurs. Cellular senescence represents a critical point of convergence within this process. Although senescence can restrict the proliferation of damaged or oncogenically stressed cells, persistent senescent cells remain metabolically active and can alter their surroundings through the senescence-associated secretory phenotype (SASP). Here, we propose the concept of a geroncogenic shift to describe how age-associated changes in tissue homeostasis may create stress-response states that are subsequently exploited by malignant and stromal cells. We distinguish two named, complementary components of the framework: systemic decline, referring to the progressive deterioration of cellular and tissue homeostasis during aging, and pathway-specific hijacking, referring to the selective co-option of altered stress-response pathways under oncogenic or therapeutic pressure. Cellular senescence provides a functional interface between these processes because the same stress responses that constrain damaged cells can, when persistent or incompletely resolved, contribute to tumor adaptation. In ovarian cancer, senescence is not a uniform cellular endpoint. Its consequences vary according to the initiating stress, cellular compartment, molecular program, duration, and capacity for recovery or escape. Tumor-cell senescence may suppress proliferation yet also be associated with stem-like adaptation and therapeutic persistence. Senescence-associated changes in fibroblasts and other stromal populations can reshape inflammatory signaling and treatment response, while polyploid and senescence-like states illustrate the extent of cellular plasticity that can exist after therapeutic stress. The SASP further extends the effects of senescence beyond individual cells by influencing neighboring tumor and stromal populations, extracellular-matrix remodeling, inflammation, and dissemination. This perspective has important therapeutic implications. Rather than treating senescence as either a uniformly protective or pathogenic state, therapeutic strategies should distinguish between senescent-cell elimination, SASP modulation, metabolic vulnerabilities, and prevention of senescence escape. We propose that understanding the cellular context and temporal dynamics of senescence will be essential for determining when senescence should be induced, maintained, modulated, or eliminated in ovarian cancer. Most therapeutic evidence for senescence-directed strategies remains preclinical, and clinical translation will require cell-type-specific biomarkers and optimized treatment timing. The geroncogenic shift framework therefore provides a conceptual basis for linking aging biology with tumor evolution and for developing more selective approaches to senescence-directed therapy.

## 1 Introduction

Ovarian cancer remains one of the most lethal gynecological malignancies. Its clinical burden reflects the frequency of advanced-stage presentation, extensive intraperitoneal dissemination, and the development of resistance after an initial response to systemic therapy (Sung et al., 2021; Webb and Jordan, 2024; Kuroki and Guntupalli, 2020). Cytoreductive surgery and platinum-based chemotherapy remain central to treatment, while PARP inhibitors and other targeted approaches have expanded therapeutic options. Nevertheless, recurrent disease remains common, and acquired resistance continues to limit durable disease control (Webb and Jordan, 2024; Kuroki and Guntupalli, 2020).

Age is one of the strongest epidemiological determinants of ovarian cancer, but chronological age alone provides an incomplete explanation for this association. Aging progressively changes the cellular and tissue environment in which malignant transformation occurs. The hallmarks of aging encompass genomic instability, telomere attrition, epigenetic alterations, deregulated nutrient sensing, mitochondrial dysfunction, cellular senescence, stem-cell exhaustion, and altered intercellular communication (López-Otín et al., 2023). Recent analyses of ovarian aging further indicate that genomic damage, telomere dysfunction, epigenetic remodeling, impaired autophagy, mitochondrial dysfunction, oxidative stress, and chronic inflammation form an interconnected biological system that progressively alters ovarian tissue homeostasis (Wu et al., 2025).

This relationship is particularly relevant to high-grade serous ovarian carcinoma (HGSOC), the predominant and most lethal epithelial subtype and the main focus of this review (Webb and Jordan, 2024; Cancer Genome Atlas Research Network, 2011). HGSOC is characterized by near-universal TP53 alteration and extensive genomic instability, with homologous recombination deficiency (HRD) representing an important feature in a subset of tumors (Cancer Genome Atlas Research Network, 2011). HR-deficient and HR-proficient tumors therefore provide distinct DNA-repair contexts in which accumulated DNA damage and therapeutic stress may interact with senescence pathways. Because the strongest evidence relevant to the present framework centers on HGSOC, studies using broader epithelial ovarian cancer models are included where they provide mechanistic information, but their findings are not assumed to be HGSOC-specific. These abnormalities provide a biological context in which accumulated DNA damage and defective repair may facilitate malignant evolution. At the same time, persistent DNA damage can activate tumor-suppressive checkpoints and restrict proliferation (Gorgoulis et al., 2019; Huang et al., 2022). The biological consequence therefore depends on whether damaged cells undergo durable arrest, are eliminated, or acquire the capacity to bypass these restraints.

Telomere dysfunction provides a particularly clear example of this duality. Stress-induced premature senescence has been implicated in ovarian tumorigenesis, and primary ovarian epithelial cells can undergo spontaneous senescence through mechanisms associated with persistent cellular stress (Raghuram and Mishra, 2014; Pakuła et al., 2020). Experimental models have further shown that telomere-mediated mitotic abnormalities in immortalized ovarian epithelial cells can reproduce chromosomal losses and breakpoints observed in ovarian carcinoma (Gisselsson et al., 2005). Telomere dysfunction can therefore contribute to tumor suppression by limiting proliferation while simultaneously creating genomic instability when cells escape the associated checkpoint.

Epigenetic remodeling provides another connection between aging and ovarian cancer. Age-associated changes in DNA methylation, histone modification, chromatin organization, and regulatory RNA networks can alter transcriptional programs involved in DNA repair, proliferation, metabolism, and cellular stress responses (López-Otín et al., 2023; Thi et al., 2024). In ovarian cancer, epigenetic alterations can affect tumor-suppressive and DNA-repair programs, although the extent to which these changes directly determine senescence remains incompletely defined (Thi et al., 2024). The relevance to senescence lies in the requirement for extensive chromatin and transcriptional remodeling during the establishment and maintenance of stable growth arrest (Gorgoulis et al., 2019; Huang et al., 2022).

Mitochondrial dysfunction adds a metabolic dimension to this relationship. Aging is accompanied by alterations in mitochondrial quality control, oxidative phosphorylation, reactive oxygen species production, and mitochondrial signaling (López-Otín et al., 2023; Wu et al., 2025). In ovarian cancer, mitochondrial alterations can support proliferation, invasion, metabolic adaptation, and resistance to therapeutic stress (Li et al., 2025). Persistent mitochondrial stress may also reinforce oxidative damage and DNA-damage signaling, thereby contributing to senescence. Conversely, tumor cells that survive prolonged metabolic stress may acquire adaptations that permit continued survival despite persistent cellular injury.

Inflammation represents a further point of convergence. Chronic inflammatory signaling associated with aging can alter tissue architecture and immune surveillance, while inflammatory pathways within ovarian tumors contribute to tumor growth, stromal activation, and immune remodeling (Liu et al., 2025; Momeny et al., 2018; Amer et al., 2025). NF-κB signaling has been implicated in the survival of chemoresistant ovarian carcinoma cells, whereas IL-6 is an important component of the inflammatory ovarian tumor microenvironment (Momeny et al., 2018; Amer et al., 2025). These observations are relevant to senescence because persistent cellular stress can activate inflammatory pathways, while senescent cells can subsequently amplify inflammatory signaling through the SASP (Coppé et al., 2010; Takasugi et al., 2022; Rossiello et al., 2022; Chien et al., 2011; Vaughan and Jat, 2011; Laberge et al., 2015; Nacarelli et al., 2020; Veenstra et al., 2022).

Cellular senescence therefore represents more than one component of the aging phenotype. It is a cellular response to persistent stress characterized by durable proliferative arrest together with substantial changes in morphology, metabolism, chromatin organization, lysosomal activity, and intercellular signaling (Gorgoulis et al., 2019; Huang et al., 2022). SA-β-gal activity remains a widely used experimental marker, but neither β-galactosidase activity nor any single marker is sufficient to establish a senescent state (Hu et al., 2024; Dimri et al., 1995). A more rigorous assessment combines persistent growth arrest with molecular and phenotypic features appropriate to the biological context (Gorgoulis et al., 2019; Huang et al., 2022; Hu et al., 2024; Dimri et al., 1995).

The distinction is particularly important in cancer. Senescence can prevent damaged or oncogenically stressed cells from continuing to proliferate and therefore function as a tumor-suppressive barrier (Gorgoulis et al., 2019; Huang et al., 2022). Senescent cells, however, remain metabolically active and can produce cytokines, chemokines, growth factors, proteases, and other mediators collectively referred to as the SASP (Coppé et al., 2010; Takasugi et al., 2022). Depending on its composition and persistence, the SASP can contribute to immune surveillance and clearance or instead promote inflammation, extracellular-matrix remodeling, stem-like phenotypes, invasion, and therapeutic resistance (Coppé et al., 2010; Takasugi et al., 2022) (Figure 1).

**FIGURE 1:**
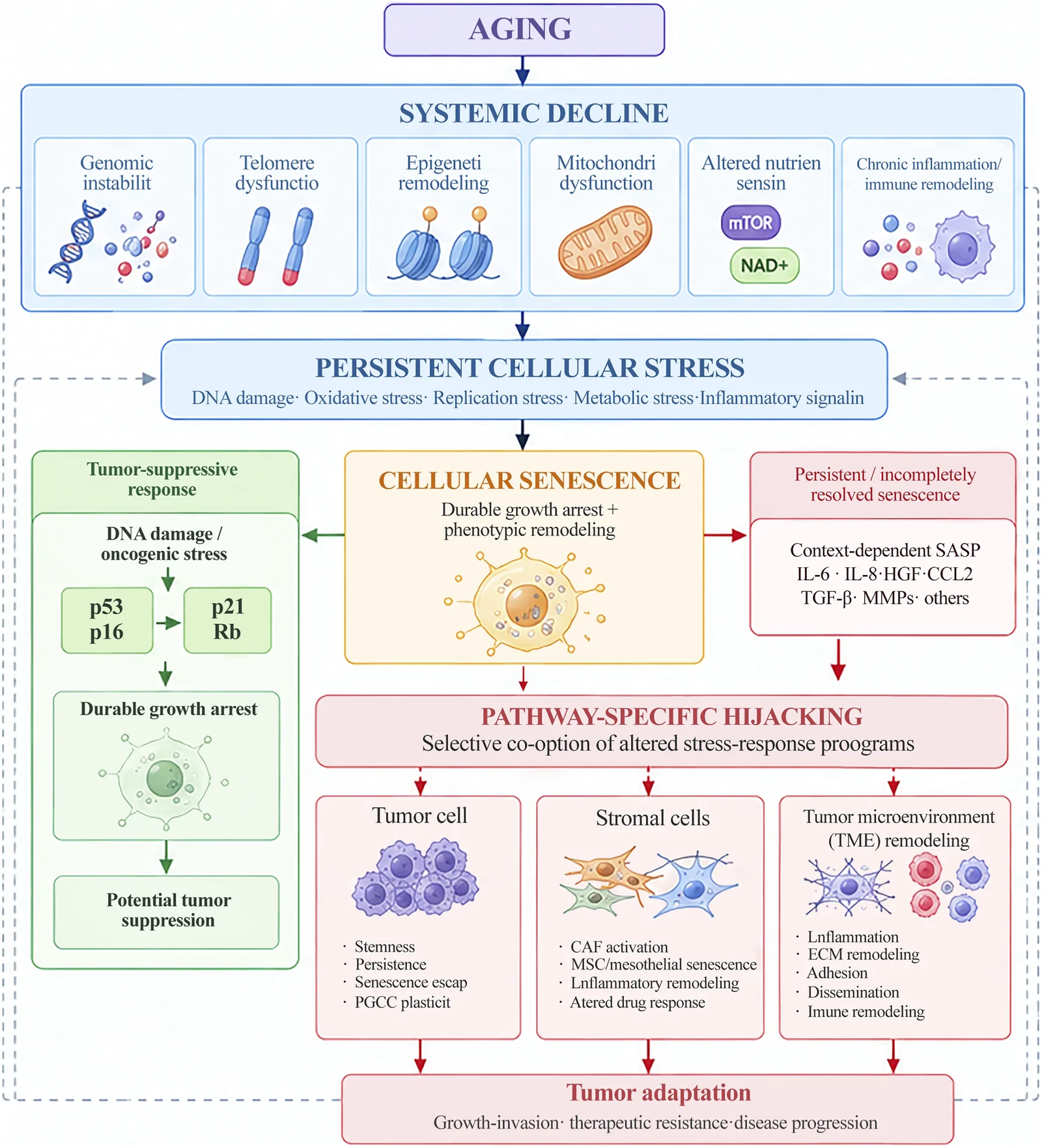
The geroncogenic shift: cellular senescence as a functional interface between aging and ovarian cancer. Aging-associated systemic decline—including genomic instability, telomere dysfunction, epigenetic remodeling, mitochondrial dysfunction, altered nutrient sensing, and chronic inflammation—converges on persistent cellular stress and cellular senescence. Senescence may restrain tumor growth through durable cell-cycle arrest or, when persistent or incompletely resolved, promote pathway-specific hijacking through context-dependent SASP signaling and remodeling of tumor cells, stromal cells, and the tumor microenvironment. In the schematic, solid lines indicate relationships supported by published mechanistic or observational evidence, whereas dashed lines indicate conceptual or indirect relationships rather than a directly demonstrated linear causal pathway. Blue denotes aging-associated inputs, green the tumor-suppressive axis, red the tumor-promoting axis, and gray dashed lines conceptual feedback relationships. The figure integrates evidence from ovarian cancer studies with findings from the broader aging and cellular-senescence literature; ovarian cancer-specific mechanisms are supported by the cited ovarian cancer studies, whereas relationships derived primarily from broader senescence or aging research are intended as contextual rather than ovarian-cancer-specific evidence.

In ovarian cancer, these opposing effects occur across multiple cellular compartments. Malignant cells can undergo therapy-induced senescence, while stromal cells may acquire senescence-associated phenotypes in response to tumor-derived or treatment-related signals (Takasugi et al., 2022; Veenstra et al., 2022; Uruski et al., 2021; Jin et al., 2023). The consequences are unlikely to be interchangeable. Tumor-cell senescence may initially restrain proliferation but subsequently influence surviving cancer cells through paracrine signaling. Stromal senescence may alter inflammatory signaling and therapeutic response, whereas senescence-associated changes within the peritoneal microenvironment may affect tumor-cell adhesion and dissemination (Veenstra et al., 2022).

The therapeutic relevance of this heterogeneity is increasingly evident. Primary HGSOC cells can undergo senescence following carboplatin–paclitaxel treatment (Uruski et al., 2021), and olaparib can induce p16-or p53-associated senescence in ovarian cancer models (Wang et al., 2019). Other ovarian cancer studies have identified additional senescence-associated therapeutic responses, including pro-senescence effects associated with IDH1 targeting and palbociclib treatment (Dahl et al., 2019; Ye et al., 2024). These observations establish therapy-induced or therapy-associated senescence as a reproducible phenomenon, but they do not imply that all senescent cells represent a permanent endpoint.

Indeed, platinum-induced senescence can be accompanied by enrichment of cancer stem-like populations through NAMPT-dependent metabolic signaling (Nacarelli et al., 2020). Polyploid giant cancer cells provide another example of cellular plasticity. Targeting PGCCs can potentiate therapeutic responses and overcome PARP inhibitor resistance in ovarian cancer models (Zhang et al., 2023), while docetaxel-induced PGCCs can display senescence-associated features together with expression of stem-cell markers (Zhao et al., 2024). These observations argue that senescence-associated phenotypes can coexist with considerable cellular plasticity.

The central question is therefore not simply whether a tumor contains senescent cells, but what those cells are doing. A senescent state may be stable or transient, tumor-cell autonomous or stromally induced, growth suppressive or tumor promoting, and potentially irreversible or capable of escape (Gorgoulis et al., 2019; Huang et al., 2022; Książek, 2022).

We use the term geroncogenic shift to describe the transition from age-associated deterioration of tissue homeostasis toward selective exploitation of aging-associated stress-response programs by malignant and stromal cells. Within this framework, systemic decline captures the progressive loss of genomic, metabolic, mitochondrial, inflammatory, and immune homeostasis accompanying aging, whereas pathway-specific hijacking captures the selective co-option of altered stress-response programs by tumor and stromal cells to survive oncogenic and therapeutic stress. Cellular senescence and the SASP occupy the functional interface between these processes (López-Otín et al., 2023; Wu et al., 2025; Gorgoulis et al., 2019; Huang et al., 2022; Coppé et al., 2010; Takasugi et al., 2022).

## 2 Hallmarks of aging in ovarian cancer

### 2.1 Genomic instability and telomere dysfunction

Genomic instability is a defining feature of HGSOC. TP53 alteration is nearly universal, while defects in homologous recombination repair contribute to widespread chromosomal abnormalities (Sistla et al., 2025; Cancer Genome Atlas Research Network, 2011). Aging provides a biological context in which cumulative DNA damage, replication stress, and declining repair capacity may increase the probability that damaged cells acquire or tolerate oncogenic alterations (López-Otín et al., 2023; Wu et al., 2025). Table 1 summarizes the aging-associated processes at the interface of ovarian aging and tumorigenesis.

**TABLE 1:** Aging-associated processes at the interface of ovarian aging and tumorigenesis.

Biological process	Age-associated alteration	Ovarian-specific evidence	Relationship to cellular senescence	Evidence level/references
Genomic instability	Accumulation of DNA damage and impaired genome maintenance	HGSOC: near-universal TP53 alteration; HRD occurs in a subset of tumors	Activates persistent DNA-damage responses and cell-cycle checkpoints	Direct/mechanistic OC; (López-Otín et al., 2023; Sistla et al., 2025; Cancer Genome Atlas Research Network, 2011; Gorgoulis et al., 2019; Huang et al., 2022)
Telomere dysfunction	Telomere attrition and structural abnormalities	Ovarian epithelial and immortalized ovarian models support telomere-associated senescence and chromosomal abnormalities; epidemiologic associations remain observational	Promotes replicative and stress-induced senescence	Mechanistic OC + observational risk data; (Gisselsson et al., 2005; Martinez-Delg et al., 2012; Rossiello et al., 2022)
Epigenetic remodeling	Altered DNA methylation, histone modification and chromatin organization	Ovarian cancer studies identify epigenetic dysregulation, while direct causal links to senescence remain incompletely defined	Influences establishment and maintenance of senescence	Mechanistic/indirect OC evidence; (López-Otín et al., 2023; Thi et al., 2024; Gorgoulis et al., 2019)
Mitochondrial dysfunction	Altered oxidative phosphorylation, ROS and mitochondrial quality control	Ovarian cancer models link mitochondrial alterations to proliferation and treatment adaptation; FDX2 provides an ovarian example	Reinforces oxidative and DNA-damage signaling	Mechanistic OC evidence; (López-Otín et al., 2023; Li et al., 2025; Huang et al., 2022)
Nutrient-sensing dysregulation	Altered mTOR, AMPK and NAD + signaling	Ovarian cancer studies connect NAMPT/NAD + metabolism and mTOR-related signaling with therapy-associated senescence and SASP.	Regulates senescence maintenance and SASP production	Mechanistic OC evidence; therapy-associated; (López-Otín et al., 2023; Laberge et al., 2015; Nacarelli et al., 2020)
Chronic inflammation	Persistent NF-kB, IL-6 and related signaling	Ovarian cancer models implicate NF-kB and IL-6-associated inflammatory signaling in tumor and stromal behavior	Reinforces inflammatory SASP programs	Direct/mechanistic OC evidence; (Liu et al., 2025; Momeny et al., 2018; Amer et al., 2025; Jin et al., 2023)
Immune remodeling	Altered immune surveillance and clearance	Direct ovarian evidence specifically linking immune remodeling to senescent-cell recognition/clearance remains limited in this review	Influences senescent-cell recognition and clearance	Indirect/general senescence evidence; OC validation limited; (Huang et al., 2022; Coppé et al., 2010; Takasugi et al., 2022)
Cellular senescence	Durable proliferative arrest with phenotypic remodeling	Primary HGSOC and ovarian cancer models demonstrate therapy-associated senescence or senescence-like states	Integrates multiple forms of cellular stress	Direct OC evidence for selected states; (Gorgoulis et al., 2019; Huang et al., 2022; Coppé et al., 2010; Takasugi et al., 2022; Uruski et al., 2021; Wang et al., 2019)
SASP remodeling	Cytokine, chemokine, growth-factor and matrix-regulating secretion	Ovarian studies provide examples involving NAMPT-dependent stem-like adaptation, stromal SASP, and mesothelial responses	Converts senescent cells into microenvironmental regulators	Direct OC examples; composition remains context dependent; (Coppé et al., 2010; Takasugi et al., 2022; Lopes-Paciencia et al., 2019; Nacarelli et al., 2020; Jin et al., 2023; Mikuła-Pietrasik et al., 2017)

The relationship between DNA damage and senescence is nevertheless bidirectional. Persistent damage can activate p53-dependent checkpoints and impose stable growth arrest, thereby limiting proliferation of cells carrying potentially oncogenic lesions (Gorgoulis et al., 2019; Huang et al., 2022). When these checkpoints are bypassed, however, continued proliferation in the presence of genomic damage may facilitate malignant evolution.

Telomere dysfunction provides a related mechanism. Ovarian epithelial cells can undergo stress-induced or spontaneous senescence (Raghuram and Mishra, 2014; Pakuła et al., 2020). Telomere-mediated mitotic disturbances in immortalized ovarian epithelial cells can generate chromosomal abnormalities resembling those observed in ovarian carcinoma (Gisselsson et al., 2005). Shorter telomere length has also been associated with ovarian cancer risk in familial and sporadic cases (Martinez-Delg et al., 2012), although the epidemiologic relationship is complex and does not establish a simple causal relationship between shorter telomeres and ovarian cancer risk. Telomere dysfunction therefore represents both a barrier to proliferation and a potential source of genomic instability when checkpoint control is circumvented.

### 2.2 Epigenetic remodeling

Epigenetic remodeling provides another interface between aging and ovarian cancer. Age-associated changes in DNA methylation, histone modification, chromatin organization, and regulatory RNA networks can alter transcriptional programs involved in DNA repair, proliferation, metabolism, and stress responses (López-Otín et al., 2023; Thi et al., 2024). In ovarian cancer, these alterations can affect gene-expression programs involved in proliferation and chemoresistance (Ramadoss et al., 2017). miR-34a promoter hypermethylation has been reported in various histological subtypes of ovarian cancer (Schmid et al., 2016).

Senescence itself is accompanied by extensive chromatin remodeling and transcriptional reprogramming (Gorgoulis et al., 2019; Huang et al., 2022). An aging-associated epigenetic landscape may therefore influence the threshold for senescence induction as well as the capacity of stressed cells to maintain or escape growth arrest. At present, however, much of the evidence linking specific epigenetic alterations to senescence in ovarian cancer remains mechanistic rather than clinically validated.

### 2.3 Mitochondrial dysfunction and metabolic stress

Mitochondrial dysfunction links aging to altered metabolic behavior in ovarian cancer. Changes in oxidative phosphorylation, reactive oxygen species production, mitochondrial quality control, and stress signaling can influence tumor-cell proliferation and adaptation to treatment (López-Otín et al., 2023; Wu et al., 2025; Li et al., 2025). Persistent mitochondrial stress may reinforce DNA damage and senescence signaling, whereas surviving tumor cells may undergo metabolic adaptation that permits continued viability under therapeutic pressure.

The relationship between metabolism and senescence is therefore not unidirectional. Metabolic dysfunction can contribute to the establishment of senescence, while senescent cells subsequently undergo metabolic remodeling that can alter their secretory behavior (Gorgoulis et al., 2019; Huang et al., 2022; Coppé et al., 2010; Takasugi et al., 2022). This distinction becomes particularly relevant after chemotherapy, when metabolic adaptation may influence the phenotype of surviving tumor cells (Santana-Rivera et al., 2020; Miyahara et al., 2024; Nacarelli et al., 2020). Metabolic adaptation in surviving tumor cells after chemotherapy can involve reduced enolase-1 expression and elevated intracellular glucose (Santana-Rivera et al., 2020), and iron-sulfur cluster assembly factor FDX2 has been shown to prevent senescence, apoptosis, or ferroptosis in ovarian cancer cells (Miyahara et al., 2024).

Metabolic reprogramming in ovarian cancer is heterogeneous rather than a simple shift from oxidative phosphorylation to glycolysis. Ovarian cancer cells can rely on aerobic glycolysis while retaining or increasing oxidative phosphorylation under metabolic or therapeutic stress. In ovarian cancer organoids and cell models, Aurora-A/SOX8/FOXK1 signaling promoted glycolytic programs, including HK2 and LDHA, while suppressing senescence and contributing to cisplatin resistance (Sun et al., 2020). Conversely, oxidative-phosphorylation dependence has been described in chemoresistant and stem-like ovarian cancer populations, supporting metabolic plasticity rather than a fixed bioenergetic state (Li et al., 2025).

Mitochondrial quality control also intersects with senescence through mitophagy. In ovarian cancer, the lncRNA-PART1/PHB2 axis links the mitophagy receptor PHB2 to cellular senescence and PARP inhibitor or cisplatin resistance. PART1 blockade promoted PHB2 degradation, impaired mitophagy, and was accompanied by senescence-associated phenotypes, whereas PHB2 restoration attenuated these effects (Wu et al., 2024). These findings provide an ovarian cancer-specific example in which altered mitochondrial turnover may influence the fate of therapy-stressed cells.

### 2.4 Chronic inflammation

Chronic inflammation represents another major point of convergence. Inflammatory signaling within ovarian tumors contributes to tumor growth, stromal activation, immune remodeling, and therapeutic resistance (Liu et al., 2025; Momeny et al., 2018; Amer et al., 2025). NF-κB inhibition can suppress growth and induce apoptosis in chemoresistant ovarian carcinoma cells (Momeny et al., 2018), while IL-6 has emerged as an important determinant of the inflammatory ovarian tumor microenvironment (Amer et al., 2025).

Inflammatory signaling is also closely linked to senescence. Persistent DNA damage and cellular stress can activate NF-κB and related pathways, whereas the SASP can maintain inflammatory signaling in neighboring cells (Coppé et al., 2010; Takasugi et al., 2022; Rossiello et al., 2022; Chien et al., 2011; Vaughan and Jat, 2011; Laberge et al., 2015; Nacarelli et al., 2020; Veenstra et al., 2022). The interaction between aging-associated inflammation and persistent senescence may therefore contribute to a tissue environment that favors tumor persistence.

In ovarian cancer, senescence-associated inflammatory signaling can involve mediators such as IL-6 and IL-8, while HGF is particularly relevant to tumor-conditioned senescence of peritoneal mesothelial cells (Amer et al., 2025; Veenstra et al., 2022; Mikuła-Pietrasik et al., 2017). These factors illustrate two complementary modes of signaling: autocrine loops can reinforce inflammatory and stress-response programs within senescent cells, whereas paracrine signaling can alter the phenotype of neighboring tumor, fibroblast, or mesothelial cells. The composition and functional impact of these loops remain context dependent rather than constituting a universal ovarian cancer SASP. In an ovarian cancer model of S1PR1 pathway inhibition, induction of senescence was accompanied by increased IL-6, further linking senescence-associated arrest with inflammatory secretory remodeling (Tao et al., 2023).

## 3 Cellular senescence: from tumor-suppressive barrier to dynamic cellular state

### 3.1 Defining senescence

Cellular senescence is generally characterized by durable proliferative arrest accompanied by extensive molecular and phenotypic remodeling (Gorgoulis et al., 2019; Huang et al., 2022). Oncogenic stress, exemplified by Ras-induced premature senescence, can trigger stable growth arrest (Serrano et al., 1997). The p53/p21 and p16/Rb pathways represent major components of the senescence response, but senescence is not defined by a single signaling pathway (Gorgoulis et al., 2019; Huang et al., 2022).

SA-β-gal activity remains widely used as an experimental marker (Dimri et al., 1995). Additional approaches include assessment of p16 and p21 expression, persistent DNA-damage signaling, chromatin remodeling, metabolic changes, and alterations in secretory activity (Gorgoulis et al., 2019; Huang et al., 2022). β-galactosidase-based probes can improve visualization of senescent cells, but marker positivity alone remains insufficient to establish a functional senescent state (Hu et al., 2024; Dimri et al., 1995).

This distinction is particularly important in cancer, where treatment-induced arrest, dormancy, polyploidization, and senescence-like states can share several molecular features. Accordingly, the terms senescence, senescence-like state, dormancy, and reversible treatment-induced arrest should not be used interchangeably (Gorgoulis et al., 2019; Huang et al., 2022; Książek, 2022).

To avoid conflating durable senescence with other forms of cell-cycle arrest or survival, we distinguish senescence from quiescence, dormancy, apoptosis, and PGCC-associated arrest according to arrest stability, viability, reversibility, and phenotypic remodeling (Table 2). This distinction is particularly important in ovarian cancer, where treatment-induced growth arrest and polyploidization may share senescence-associated markers without representing the same functional state (Gorgoulis et al., 2019; Huang et al., 2022; Książek, 2022; Zhang et al., 2023; Zhao et al., 2024).

**TABLE 2:** Cellular senescence states and their functional consequences in ovarian cancer.

Category/cellular compartment	State/trigger	Key evidence/features	Functional consequence	Therapeutic implication/references
Tumor-intrinsic — ovarian cancer cells	Platinum-induced senescence	DDR; p53–p21 and p16–Rb signaling; durable arrest	Initial growth suppression	Characterize persistence and selectively clear persistent cells; (Uruski et al., 2021)
Tumor-intrinsic — ovarian cancer cells	Platinum-induced senescence + NAMPT	NAMPT/NAD + metabolism; ALDH1A1-high and CD133-positive stem-like cells	Stem-like adaptation under therapy	NAMPT inhibition; (Nacarelli et al., 2020)
Tumor-intrinsic — ovarian cancer cells	PARP inhibitor-associated arrest/senescence	p16- and p53-associated arrest; reversibility is context dependent	Growth suppression; senescence is not the universal resistance mechanism	Distinguish stable senescence from reversible arrest; (Fleury et al., 2019; Wang et al., 2019)
Tumor-intrinsic — ovarian cancer cells	PGCC-associated senescence-like state	Persistent DNA-damage signaling, polyploidization and stemness-associated factors	Cellular plasticity and resistant progeny	Target PGCC formation/survival; (Zhang et al., 2023; Niu et al., 2021; Zhao et al., 2024)
Tumor-intrinsic — ovarian cancer cells	Senescence suppression (Aurora-A/SOX8/FOXK1)	Evidence supports pathway-associated/marker-level regulation; durable arrest not established here	Reduced senescence and increased chemoresistance	Context-specific pathway targeting; (Sun et al., 2020)
Tumor-intrinsic — ovarian cancer cells	S1PR1-associated senescence regulation	PDK1–LATS1/2–YAP signaling; pathway/phenotypic evidence	Modulation of proliferation and senescence	Context-specific pathway targeting; (Tao et al., 2023)
Tumor-intrinsic — ovarian cancer cells	ITPKA-associated senescence	p53 stabilization with comparatively stronger senescence-phenotype support	Senescence induction and tumor suppression	Explore restoration of p53-associated senescence; (Shaosheng et al., 2021)
Tumor-intrinsic — ovarian cancer cells	lncRNA-PART1/PHB2-associated senescence	Senescence-associated mitochondrial features accompany drug resistance; causality of senescence per se remains unproven	Association with PARP inhibitor/cisplatin resistance	Mechanistic dissection of the PART1–PHB2 axis; (Wu et al., 2024)
Stromal/peritoneal — CAFs	PARP inhibitor-induced inflammatory phenotype	NF-kB-driven SASP; ovarian CAF-specific evidence	Stromal inflammation and reduced PARP inhibitor sensitivity	SASP modulation with bepotastine; (Jin et al., 2023)
Stromal/peritoneal — mesothelial niche	Tumor-conditioned senescence	HGF-dependent, oxidative-stress-associated senescence in peritoneal mesothelial cells	Increased ovarian cancer adhesion/permissive peritoneal niche	Target the relevant local senescent niche; (Veenstra et al., 2022; Mikuła-Pietrasik et al., 2017)
Intercellular — recipient cells	Extracellular-vesicle senescence signaling	YBX1-dependent Satellite II RNA loading into sEVs; general senescence model	Propagation of senescence-associated inflammatory signaling	Not established in ovarian cancer; retain as general context; (Chiba et al., 2023)
Tumor-intrinsic — ovarian cancer cells	Hypoxia-associated senescence-like state	CSN8-associated signaling in epithelial ovarian cancer cells	Tumor-promoting senescence-like phenotype	Target CSN8-associated signaling; (Ruan et al., 2026)
Tumor-intrinsic — ovarian cancer cells	Senescence evasion	YTHDC1–m6A–TERT signaling	Escape from senescence	Restore senescence susceptibility; (Wang et al., 2026)

### 3.2 Molecular establishment and maintenance

Persistent DNA damage can activate p53/p21 signaling and reinforce cell-cycle arrest, while p16/Rb signaling provides an additional barrier to cell-cycle progression (Gorgoulis et al., 2019; Huang et al., 2022). The relative contribution of these pathways depends on cellular genotype and the initiating stress.

Senescence is also influenced by metabolic and epigenetic regulators. mTOR signaling can regulate the secretory phenotype of senescent cells, while NAD+ metabolism affects inflammatory secretory activity (Laberge et al., 2015; Nacarelli et al., 2020). NF-κB and p38 MAPK provide additional regulatory layers for SASP development (Chien et al., 2011; Vaughan and Jat, 2011; Freund et al., 2011). These pathways help determine not only how growth arrest is maintained but also how the senescent cell communicates with its surroundings.

### 3.3 Heterogeneity of senescence

The biological diversity of senescence is central to its interpretation in ovarian cancer. Tumor-suppressive senescence can prevent proliferation of damaged cells and contribute to tumor control. Therapy-induced senescence can accompany an initial response to chemotherapy or targeted therapy. Stromal cells can acquire senescence-associated inflammatory phenotypes. Senescence-like states can retain sufficient plasticity to permit subsequent cell-cycle re-entry (Gorgoulis et al., 2019; Huang et al., 2022; Książek, 2022).

The ovarian cancer studies of Aurora-A, S1PR1, ITPKA and lncRNA-PART1 illustrate this heterogeneity (Sun et al., 2020; Tao et al., 2023; Shaosheng et al., 2021; Wu et al., 2024). Aurora-A/SOX8/FOXK1 signaling can suppress senescence and promote chemoresistance, whereas ITPKA-mediated stabilization of p53 promotes senescence and restricts tumorigenesis (Sun et al., 2020; Shaosheng et al., 2021). S1PR1 regulates senescence through the PDK1–LATS1/2–YAP pathway (Tao et al., 2023). The lncRNA-PART1–PHB2 axis provides an apparently different phenotype in which increased senescence is associated with resistance to cisplatin and PARP inhibition (Wu et al., 2024).

The ovarian cancer pathway studies also reveal distinct molecular routes into or away from senescence. In the Aurora-A/SOX8/FOXK1 axis, Aurora-A directly phosphorylates SOX8 at Ser327, enhancing FOXK1 activity; this program increases glycolytic regulators such as HK2 and LDHA and supports hTERT expression while suppressing P16-associated senescence, thereby linking metabolic rewiring to cisplatin resistance (Sun et al., 2020). In the S1PR1 pathway, S1PR1 activation increases PDK1 and suppresses LATS1/2, favoring YAP activity; S1PR1 deletion or pharmacologic inhibition has the opposite effect and increases senescence-associated proteins, including P21, P27, PAI-1, and IGFBP7. YAP can transcriptionally reinforce S1PR1 expression, forming a positive-feedback circuit (Tao et al., 2023). The Aurora-A/SOX8/FOXK1 and S1PR1 studies are best described as demonstrating regulation of senescence-associated phenotypes or markers, rather than establishing a universal, durable senescent state.

ITPKA provides a more directly validated tumor-suppressive mechanism: ITPKA interacts with MDM2, reduces P53 ubiquitination, stabilizes P53, and increases P21; P53 knockdown attenuates the senescence phenotype, supporting a functional P53-dependent senescence mechanism (Shaosheng et al., 2021). By contrast, the lncRNA-PART1/PHB2 pathway connects senescence with mitochondrial quality control. PART1 interacts with PHB2 and supports its stability; PART1 inhibition impairs mitophagy, promotes senescence-associated changes, and increases PARP inhibitor and cisplatin resistance, while PHB2 restoration reduces these phenotypes (Wu et al., 2024). Additional pathway-level examples include progesterone receptor-B signaling, which induces FOXO1-dependent p21 expression and senescence, and miR-506, which directly targets CDK4/CDK6 to suppress the CDK4/6-FOXM1 axis and promote senescence with little evidence of apoptosis as the dominant mechanism (Diep et al., 2013; Liu et al., 2014). These findings are not necessarily contradictory. They indicate that the biological value of senescence to a tumor depends on the particular state induced, the cellular compartment involved, and the consequences of maintaining or escaping that state (Gorgoulis et al., 2019; Huang et al., 2022; Książek, 2022). The ITPKA study provides stronger evidence for a p53-associated senescence phenotype, whereas the lncRNA-PART1/PHB2 study links senescence-associated features with drug resistance but does not establish that senescence itself is the primary cause of resistance.

## 4 The SASP and tumor-microenvironment remodeling

The SASP is not a fixed collection of cytokines. Its composition depends on the initiating stimulus, cellular identity, duration of senescence, and intracellular signaling environment (Coppé et al., 2010; Takasugi et al., 2022; Lopes-Paciencia et al., 2019). Proteomic atlases have begun to catalog the diversity of senescence-associated secretomes (Basisty et al., 2020).

Examples relevant to ovarian cancer include IL-6 and IL-8 among inflammatory mediators and HGF as a growth-factor signal implicated in tumor–peritoneal stromal communication. These molecules should be regarded as context-dependent components rather than as a universal SASP signature, because their abundance and biological effects vary with cell identity, inducing stress, and duration of senescence (Amer et al., 2025; Coppé et al., 2010; Takasugi et al., 2022; Lopes-Paciencia et al., 2019; Mikuła-Pietrasik et al., 2017).

NF-κB is an important regulator of inflammatory SASP programs (Chien et al., 2011; Vaughan and Jat, 2011). PRMT1 is recruited to chromatin and sustains the senescence-associated secretory phenotype in response to cisplatin (M et al., 2020). mTOR contributes to SASP production through effects on protein synthesis and IL-1α translation (Laberge et al., 2015). p38 MAPK provides an additional regulatory layer (Freund et al., 2011). NAD + metabolism also influences inflammatory secretory activity (Nacarelli et al., 2020). These pathways should therefore be viewed as components of a broader regulatory network rather than as independent master pathways.

The consequences of SASP signaling are similarly context dependent. Secretory activity can facilitate immune recognition and clearance of senescent cells, but persistent SASP can also promote inflammation and alter the behavior of neighboring cells (Coppé et al., 2010; Takasugi et al., 2022).

The composition and functional consequences of the SASP are highly context dependent, varying with cell identity, the nature of the inducing stress, and the duration of senescence (Coppé et al., 2010; Takasugi et al., 2022; Lopes-Paciencia et al., 2019). Ovarian cancer-specific studies are therefore particularly informative for defining how senescence-associated signaling shapes tumor progression and therapeutic response. Ovarian cancer-specific studies have also demonstrated that tumor-derived signals can induce senescence-associated phenotypes in stromal cells, thereby altering the behavior of the peritoneal microenvironment (Mikuła-Pietrasik et al., 2017).

General senescence studies support the capacity of the SASP to influence neighboring-cell behavior (Coppé et al., 2010; Takasugi et al., 2022). In ovarian cancer, NAMPT-dependent stem-like adaptation and stromal SASP-mediated therapeutic resistance provide direct examples (Nacarelli et al., 2020; Jin et al., 2023).

In an ovarian cancer-specific study, Mikuła-Pietrasik et al. showed that conditioned medium from ovarian cancer cells induced an HGF-dependent, oxidative-stress-associated senescence phenotype in human peritoneal mesothelial cells. These senescent mesothelial cells subsequently promoted ovarian cancer cell adhesion, providing a direct link between tumor-derived signals, stromal senescence, and the formation of a permissive peritoneal microenvironment (Mikuła-Pietrasik et al., 2017).

### 4.1 SASP and cancer stemness

A key ovarian cancer study by Nacarelli et al. showed that platinum-induced senescence was associated with enrichment of cancer stem-like cells characterized by increased ALDH1A1 activity and CD133 expression. Inhibition of NAMPT suppressed this phenotype and improved cisplatin response in experimental ovarian cancer models (Nacarelli et al., 2020).

This finding is important because it suggests that therapy-induced senescence can influence the phenotype of surviving tumor cells rather than simply preventing their proliferation. NAMPT-dependent metabolic remodeling may therefore represent a functional determinant of the consequences of therapy-induced senescence (Nacarelli et al., 2020).

### 4.2 Extracellular vesicle-mediated communication

YBX1 has been reported to regulate the loading of Satellite II RNA into small extracellular vesicles, with these vesicles capable of promoting senescence-associated phenotypes in recipient cells (Chiba et al., 2023). Although this mechanism has not yet been demonstrated in ovarian cancer, it illustrates a conserved route of senescence-associated intercellular communication that may also operate in the ovarian tumor microenvironment.

### 4.3 Peritoneal microenvironment

The peritoneal microenvironment is particularly relevant to ovarian cancer because transcoelomic dissemination depends on interactions among tumor cells, mesothelial cells, fibroblasts, extracellular matrix, and ascitic factors. Senescence-associated signaling may contribute to this process by altering the behavior of non-malignant cells within the peritoneal niche (Veenstra et al., 2022).

Mikuła-Pietrasik et al. showed that conditioned medium from ovarian cancer cells induces HGF-dependent, oxidative-stress-associated senescence in human peritoneal mesothelial cells. Importantly, the resulting senescent mesothelial cells promoted ovarian cancer cell adhesion, suggesting that tumor-induced stromal senescence can contribute directly to the establishment of a permissive metastatic niche (Mikuła-Pietrasik et al., 2017). Recent work further indicates that chemotherapy-induced SASP can promote cell detachment and metastatic dissemination through metabolic reprogramming (Cole et al., 2026).

These findings place senescence within the broader biology of peritoneal dissemination rather than restricting its effects to the malignant epithelial compartment. However, the available evidence does not justify assuming that all mesothelial or stromal senescence has the same consequences; the initiating stimulus, cellular context, and composition of the associated secretory program remain important determinants.

These three compartments should therefore be distinguished mechanistically. Malignant-cell senescence is commonly initiated by intrinsic oncogenic or genotoxic stress, including chemotherapy or PARP inhibition. CAF-associated senescence or inflammatory phenotypes are more strongly shaped by tumor-derived and treatment-related signals and can alter drug response through SASP programs. Peritoneal mesothelial senescence, in contrast, has been directly linked in ovarian cancer models to tumor-conditioned, HGF-dependent oxidative stress and to increased tumor-cell adhesion. Thus, “stromal senescence” is not a single biological entity, and its interpretation should remain compartment specific (Veenstra et al., 2022; Jin et al., 2023; Mikuła-Pietrasik et al., 2017).

## 5 Senescence and therapeutic response

Therapy-induced senescence can initially contribute to tumor control. Primary HGSOC cells are susceptible to senescence induced by carboplatin and paclitaxel (Uruski et al., 2021), and olaparib can induce p16-or p53-associated senescence in ovarian cancer models (Wang et al., 2019). Senescence induction has also been shown to dictate responses to chemotherapy and immunotherapy in preclinical ovarian cancer models (Paffenholz et al., 2022). Additional ovarian cancer studies have demonstrated pro-senescence effects associated with IDH1 targeting and palbociclib treatment (Dahl et al., 2019; Ye et al., 2024). Table 3 summarizes the heterogeneity of senescence-associated states across different cellular compartments in ovarian cancer.

**TABLE 3:** Distinguishing senescence from other forms of growth arrest and cell fate in ovarian cancer.

State/fate	Arrest and viability	Reversibility/escape	Key distinguishing features	Interpretation in ovarian cancer
Cellular senescence	Durable arrest; viable and metabolically active	Usually stable, but escape may occur in some cancer contexts	Persistent arrest plus molecular/phenotypic remodeling; no single marker is diagnostic	Requires multidimensional validation; may suppress growth yet support SASP-mediated adaptation
Quiescence	Reversible arrest; viable	Readily reversible after appropriate signals return	Reduced proliferation without the full senescence remodeling program	Reduced proliferation alone should not be labeled senescence
Dormancy	Prolonged or reversible non-proliferative state; viable	Potential re-entry into the cell cycle	Often stress- or niche-dependent; can involve residual tumor cells	Relevant to residual disease but distinct from canonical senescence
Apoptosis	Cell death; non-viable at completion	Irreversible	Programmed cell death with apoptotic execution features	Distinct treatment outcome even when triggered by the same genotoxic stress
PGCC-associated arrest	Profound arrest/atypical cycling; often viable	Variable; plasticity and progeny generation reported	Polyploidization, enlarged cells, persistent stress and stemness-associated features	Best described as senescence-like unless durable senescence is independently demonstrated

These studies establish senescence or senescence-associated phenotypes as treatment-associated cellular responses. They do not, however, establish that senescence itself is synonymous with therapeutic resistance.

The distinction becomes important when senescent cells persist after treatment.

### 5.1 NAMPT-dependent stem-like states

Platinum-induced senescence can be accompanied by a stem-like phenotype in ovarian cancer. Nacarelli et al. demonstrated that NAMPT inhibition suppresses cancer stem-like cells associated with therapy-induced senescence and improves cisplatin response (Nacarelli et al., 2020). The study provides direct evidence that metabolic regulation can influence the consequences of therapy-induced senescence.

This observation is more accurately described as a senescence-associated state transition than as a simple “senescence-mediated resistance” mechanism. The surviving tumor population appears to acquire a phenotype with enhanced stem-like characteristics under therapeutic stress (Nacarelli et al., 2020).

### 5.2 Polyploid giant cancer cells

PGCCs provide an additional example of senescence-associated cellular plasticity. Zhang et al. demonstrated that targeting PGCCs can potentiate treatment response and overcome resistance to PARP inhibitors in ovarian cancer models (Zhang et al., 2023). The study supports the concept that PGCCs represent a therapeutically relevant population associated with treatment resistance.

Independent work by Zhao et al. showed that docetaxel-induced PGCCs in ovarian cancer display senescence-associated features together with expression of stem-cell markers (Zhao et al., 2024). These findings suggest that PGCCs can occupy a cellular state in which senescence-associated phenotypes coexist with developmental plasticity. Cisplatin resistance can also be mediated through cellular senescence via the NACC1/GADD45GIP1 axis (Nakayama et al., 2017).

IL-6 provides another layer of regulation. Niu et al. demonstrated that IL-6 promotes drug resistance through PGCC formation and stromal fibroblast reprogramming (Niu et al., 2021). This finding links inflammatory signaling with both tumor-cell plasticity and stromal remodeling.

### 5.3 PARP inhibitor-associated senescence and inflammatory signaling

PARP inhibition can interact with cellular senescence in a context-dependent manner. Experimental studies have demonstrated an interplay between PARP inhibition and reversible cancer-cell senescence, suggesting that treatment-induced growth arrest may represent a dynamic state rather than an irreversible endpoint (Fleury et al., 2019). In ovarian cancer, olaparib has been shown to induce p16-or p53-associated senescence (Wang et al., 2019), while senescence-related mechanisms have also been linked to PARP inhibitor resistance through the lncRNA-PART1–PHB2 axis and PGCC-associated cellular plasticity (Wu et al., 2024; Zhang et al., 2023).

Mechanistically, PARP inhibition imposes replication-associated DNA damage and can induce senescence in ovarian cancer models, with the response influenced by homologous-recombination status. Senescence should therefore be viewed as one possible cellular response to PARP inhibition rather than as the primary or universal mechanism of PARP inhibitor resistance (Cancer Genome Atlas Research Network, 2011; Wu et al., 2024; Fleury et al., 2019; Wang et al., 2019).

## 6 Stromal senescence and therapeutic resistance

Therapy-induced senescence is not restricted to malignant cells. Stromal contributions to therapy resistance are not limited to CAFs; mesenchymal stromal cells can also secrete soluble factors that confer platinum chemoresistance to ovarian cancer cells (Koren Carmi et al., 2023). Cancer-associated fibroblasts can also acquire inflammatory phenotypes after therapeutic exposure.

Jin et al. demonstrated that PARP inhibition induces an NF-κB-dependent SASP in ovarian cancer-associated fibroblasts and that bepotastine suppresses this response, thereby increasing ovarian cancer sensitivity to PARP inhibition (Jin et al., 2023). This provides direct ovarian cancer evidence that stromal SASP can contribute to therapeutic resistance.

This observation changes the interpretation of therapy-induced senescence. A treatment may effectively arrest malignant cells while simultaneously altering stromal signaling in a manner that supports residual disease. The relevant biological unit is therefore not necessarily the tumor cell alone, but the interaction between tumor cells and the surrounding microenvironment (Takasugi et al., 2022; Jin et al., 2023).

Ruan et al. further identified a hypoxia-associated senescence-like state driven by CSN8 in epithelial ovarian cancer cells (Ruan et al., 2026). Wang et al. reported that YTHDC1 promotes senescence evasion through m6A-mediated TERT stabilization (Wang et al., 2026). These studies broaden the spectrum of senescence-related states in ovarian cancer, but their findings should be interpreted according to the specific cellular model and mechanism investigated rather than generalized to all forms of senescence.

## 7 Therapeutic strategies targeting cellular senescence

The current evidence supports several distinct therapeutic approaches, each directed at a different component of the senescence program (Figure 2). Most ovarian cancer studies discussed in this section are preclinical, and clinical observations are noted where available without establishing senescence-directed efficacy.

**FIGURE 2:**
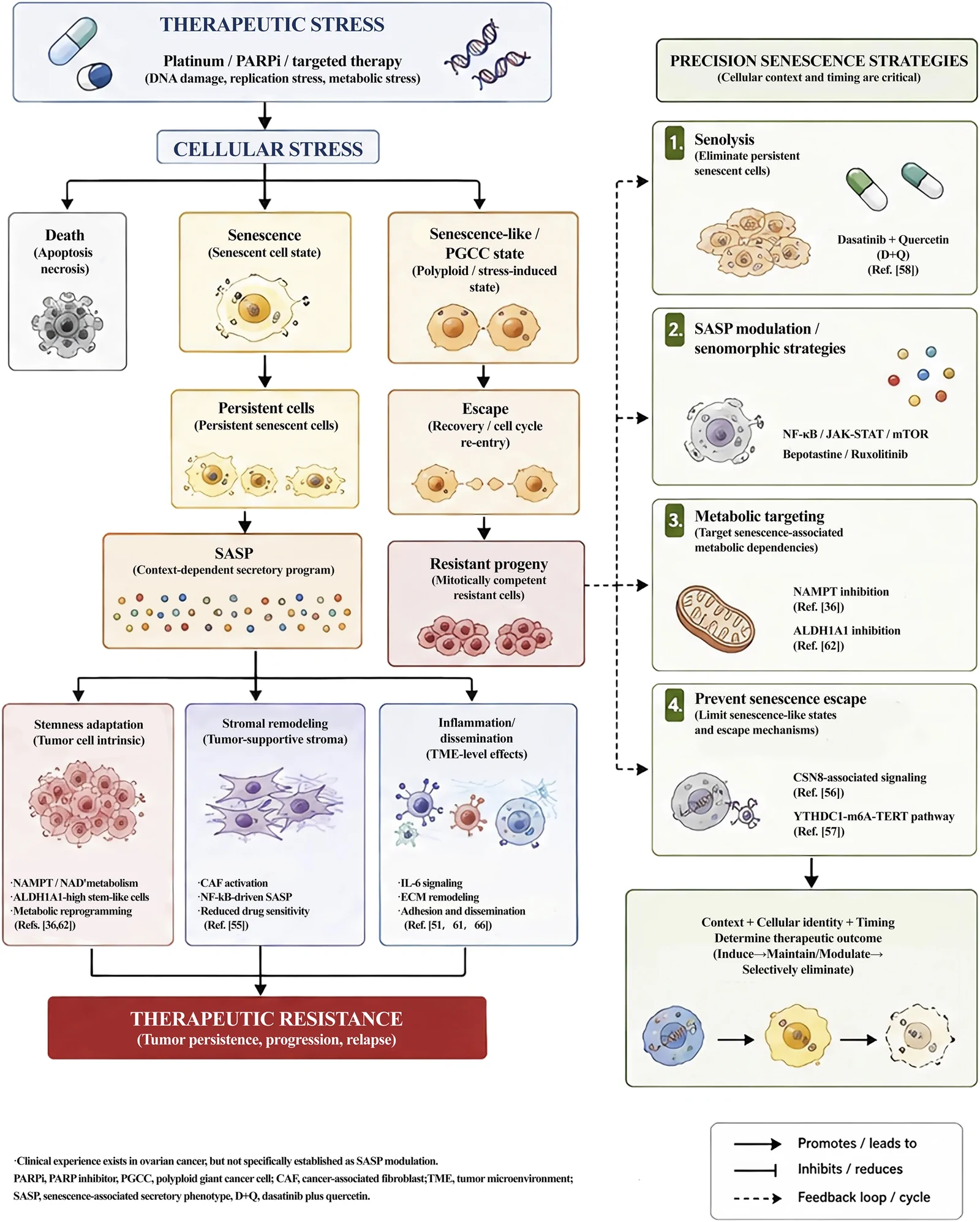
Therapy-induced senescence, cellular plasticity, and precision senescence-directed strategies in ovarian cancer. Therapeutic stress can induce diverse cellular fates, including cell death, senescence, and senescence-like or polyploid states. Persistent senescence and senescence-associated plasticity may contribute to stem-like adaptation, stromal remodeling, inflammation, dissemination, and therapeutic resistance. Potential interventions include senolysis, SASP modulation, metabolic targeting, and prevention of senescence escape, with therapeutic outcomes determined by cellular context and treatment timing. In the schematic, solid arrows indicate documented cellular-state transitions or mechanistic relationships supported by published evidence, whereas dashed connectors from biological states to therapeutic strategies indicate candidate intervention points rather than established therapeutic efficacy. Most therapeutic examples shown are supported by preclinical ovarian cancer studies. Ruxolitinib represents clinical experience in ovarian cancer but was not developed as a senescence-specific or SASP-specific therapy. The figure incorporates recent ovarian cancer studies describing hypoxia-associated CSN8-mediated senescence-like states, YTHDC1-mediated senescence evasion, and chemotherapy-induced SASP-associated dissemination (Ruan et al., 2026; Wang et al., 2026; Cole et al., 2026).

### 7.1 Senolysis

Dasatinib combined with quercetin has shown preclinical activity in ovarian cancer models. Wang et al. reported that combining this senolytic regimen with carboplatin or olaparib reduced peritoneal and adipose-tissue metastasis (Wang et al., 2024). These findings support the therapeutic potential of senolysis in ovarian cancer, but they remain preclinical and do not establish clinical efficacy.

The relevant therapeutic question is therefore not simply whether senescent cells can be eliminated, but whether the specific senescent population being targeted contributes to tumor persistence.

### 7.2 SASP modulation and senomorphic strategies

SASP modulation represents an alternative to direct elimination of senescent cells. mTOR signaling is a recognized regulator of SASP production (Laberge et al., 2015), while NAD + metabolism can influence inflammatory secretory activity (Nacarelli et al., 2020). Ruxolitinib provides clinical experience with JAK inhibition in advanced ovarian cancer, although the available clinical study was not designed to establish SASP modulation as its mechanism of action (Landen et al., 2024).

Senolytic and senomorphic strategies should be distinguished by their therapeutic target. Senolytics aim to selectively eliminate viable senescent cells, exemplified here by dasatinib plus quercetin, whereas senomorphic strategies suppress harmful senescence-associated functions without necessarily removing the cells. In this framework, NF-κB, JAK/STAT, and mTOR-directed interventions are best considered senomorphic or SASP-modulating approaches when their principal effect is suppression of inflammatory or secretory signaling; their activity should not automatically be equated with senescent-cell clearance (Laberge et al., 2015; Jin et al., 2023).

Bepotastine provides a stronger example. Jin et al. showed that bepotastine suppresses NF-κB-triggered SASP in ovarian cancer-associated fibroblasts and sensitizes ovarian cancer to PARP inhibition (Jin et al., 2023).

### 7.3 Metabolic targeting

NAMPT inhibition represents a particularly informative strategy because it targets a metabolic dependency associated with therapy-induced senescence rather than eliminating senescent cells indiscriminately. Nacarelli et al. showed that NAMPT inhibition suppresses the stem-like population associated with therapy-induced senescence and improves cisplatin response (Nacarelli et al., 2020).

ALDH1A1 inhibition provides another ovarian cancer-specific strategy. Muralikrishnan et al. reported that a novel ALDH1A1 inhibitor blocks platinum-induced senescence and stemness in ovarian cancer models (Muralikrishnan et al., 2022). Pro-senescence therapy has also been explored through targeting ribonucleotide reductase M2 in epithelial ovarian cancer (Aird et al., 2014). Together, these studies suggest that senescence-associated metabolic dependencies may provide more selective therapeutic vulnerabilities than senescence itself.

### 7.4 Preventing senescence escape

The recent CSN8 and YTHDC1 studies suggest that preventing tumor-promoting senescence-like states or limiting escape from senescence may represent another therapeutic strategy (Ruan et al., 2026; Wang et al., 2026). Comparative analyses indicate that spontaneous and drug-induced senescence can exert pro-tumorigenic effects in ovarian cancer models (Rutecki et al., 2022).

This distinction is important. Senescence induction, senescence maintenance, SASP production, senescent-cell survival, and senescence escape are biologically different processes. A drug that affects one component should not automatically be interpreted as a general senescence-targeting agent.

### 7.5 Combination strategies

NF-κB inhibition provides an example of inflammatory pathway targeting in chemoresistant ovarian cancer. Momeny et al. showed that NF-κB blockade inhibits growth and induces apoptosis in chemoresistant ovarian carcinoma cells (Momeny et al., 2018). Dasatinib plus quercetin combined with carboplatin or olaparib provides a separate preclinical example of combining conventional treatment with senolysis (Wang et al., 2024).

A sequential strategy in which therapy-induced senescence is followed by selective clearance of persistent senescent cells is biologically attractive, but its optimal timing, safety, and clinical efficacy remain to be established. Clinical translation is further limited by the difficulty of distinguishing tumor-promoting senescent cells from senescent populations with tissue-protective functions and by the potential toxicity of non-selective senescence-targeted interventions. Biomarker-guided patient selection and treatment sequencing will therefore be essential before these approaches can be evaluated clinically.

## 8 Discussion

The central argument of this review is that cellular senescence occupies a mechanistic interface between aging and ovarian cancer rather than representing an isolated biological process.

Aging-associated genomic instability, telomere dysfunction, epigenetic remodeling, mitochondrial stress, altered nutrient sensing, and chronic inflammation progressively reshape tissue homeostasis (López-Otín et al., 2023; Wu et al., 2025). These changes can increase cellular stress and alter the threshold at which damaged cells enter senescence. In parallel, malignant cells acquire mechanisms that allow them to bypass, exploit, or escape these stress responses (Sistla et al., 2025; Cancer Genome Atlas Research Network, 2011; Gorgoulis et al., 2019; Huang et al., 2022).

Senescence therefore has several possible biological consequences. A damaged cell may enter stable arrest and contribute to tumor suppression. A therapy-treated tumor cell may enter senescence but subsequently influence surviving cells through metabolic or secretory signaling. A stromal cell may acquire an inflammatory phenotype that changes therapeutic sensitivity. Alternatively, a senescence-like state may retain sufficient plasticity to permit subsequent proliferation (Gorgoulis et al., 2019; Huang et al., 2022; Coppé et al., 2010; Takasugi et al., 2022).

The ovarian cancer literature provides examples of each possibility. Aurora-A/SOX8/FOXK1 signaling suppresses senescence and contributes to chemoresistance (Sun et al., 2020), whereas ITPKA promotes p53-associated senescence and suppresses tumorigenesis (Shaosheng et al., 2021). NAMPT-dependent signaling associates therapy-induced senescence with stem-like populations (Nacarelli et al., 2020). PGCCs illustrate the coexistence of senescence-associated features with cellular plasticity (Zhang et al., 2023; Zhao et al., 2024). CAF-associated SASP can contribute to PARP inhibitor resistance (Jin et al., 2023).

These observations support the concept of a geroncogenic shift. The term does not imply that aging itself directly causes ovarian cancer. Rather, it describes a change in biological context in which age-associated deterioration creates altered stress-response landscapes that malignant cells and stromal populations can selectively exploit.

### 8.1 Senescence as a spectrum of functional states

The available evidence is more consistent with a spectrum of senescence-associated states than with a binary distinction between senescent and non-senescent cells (Gorgoulis et al., 2019; Huang et al., 2022; Książek, 2022).

At one end, durable growth arrest may provide effective tumor suppression. At another, persistent SASP-producing cells may support inflammation and tumor adaptation. Between these states lie therapy-induced senescence, senescence-like states, dormancy, and polyploid populations with variable capacity for recovery (Gorgoulis et al., 2019; Huang et al., 2022; Książek, 2022; Zhang et al., 2023; Zhao et al., 2024).

This spectrum has direct implications for interpretation of experimental data. A positive SA-β-gal signal does not establish irreversibility. Likewise, p16 or p21 expression alone does not determine whether a cell is functionally senescent. This is why multidimensional characterization is essential (Gorgoulis et al., 2019; Huang et al., 2022).

### 8.2 Tumor-cell and stromal senescence are not equivalent

Tumor-cell senescence and stromal senescence should not be treated as interchangeable biological phenomena.

In tumor cells, senescence can restrict proliferation but may also be associated with stem-like adaptation (Nacarelli et al., 2020). In CAFs, senescence-associated inflammatory signaling can alter response to PARP inhibition (Jin et al., 2023). In the ovarian cancer dissemination context, the SASP has been implicated in interactions between senescent cells and the tumor microenvironment (Veenstra et al., 2022).

The therapeutic consequence is that senescence-directed interventions should ideally be guided by cellular identity and function rather than by a generic senescence marker.

### 8.3 The importance of temporal control

The timing of senescence-directed treatment is likely to be critical.

Eliminating a senescent population immediately after therapy may have different consequences from eliminating persistent cells after a prolonged period of SASP production. Similarly, suppressing SASP without eliminating the underlying cells may be useful in one context but insufficient in another (Coppé et al., 2010; Takasugi et al., 2022).

Current preclinical studies support the feasibility of senolysis (Wang et al., 2024), SASP modulation (Jin et al., 2023), metabolic targeting (Nacarelli et al., 2020; Muralikrishnan et al., 2022), and prevention of senescence escape (Ruan et al., 2026; Wang et al., 2026). None yet establishes a universal therapeutic sequence for ovarian cancer.

### 8.4 Toward precision senescence modulation

The field should therefore move beyond the broad question of whether senescence should be induced or eliminated. A more informative framework asks which cells are senescent, what initiated the state, whether the arrest is stable, which secretory program is active, and whether the state contributes to tumor control or tumor persistence (Gorgoulis et al., 2019; Huang et al., 2022; Coppé et al., 2010; Takasugi et al., 2022).

This distinction is particularly important for ovarian cancer because the disease is anatomically and biologically heterogeneous. Tumor cells, fibroblasts, mesothelial cells, immune cells, and other stromal populations can experience different stresses within the same tumor (Takasugi et al., 2022; Veenstra et al., 2022; Jin et al., 2023).

Future studies should therefore integrate cellular identity, spatial localization, senescence-associated molecular profiles, SASP composition, and treatment history. Such approaches may allow senescence-directed therapies to be deployed selectively rather than indiscriminately.

## 9 Conclusion

Cellular senescence provides a conceptual link between age-associated stress, ovarian cancer plasticity, and therapeutic response, but current evidence supports a context-dependent rather than uniform role.

Major gaps remain in defining the cellular and tissue origin of senescent populations; standardizing multidimensional phenotyping beyond single markers; resolving spatial and temporal heterogeneity across tumor, peritoneal, and stromal compartments; directly validating reversibility and senescence escape; developing cell-type- and state-specific biomarkers; and determining how induction, maintenance, SASP modulation, and senolysis should be scheduled relative to treatment.

Most therapeutic evidence remains preclinical. Progress toward clinical translation will require biomarker-linked, longitudinal studies that determine which senescence-associated states should be preserved, modulated, or selectively eliminated in specific ovarian cancer contexts.
